# Capacity building of healthcare workers: Key step towards elimination of viral hepatitis in developing countries

**DOI:** 10.1371/journal.pone.0253539

**Published:** 2021-06-24

**Authors:** Aayushi Rastogi, Sapna Chauhan, Archana Ramalingam, Madhavi Verma, Seena Babu, Sarita Ahwal, Akanksha Bansal

**Affiliations:** 1 Department of Epidemiology, Institute of Liver and Biliary Sciences, New Delhi, Delhi, India; 2 Project ECHO & PRAKASH, Institute of Liver and Biliary Sciences, New Delhi, Delhi, India; 3 College of Nursing, Institute of Liver and Biliary Sciences, New Delhi, Delhi, India; 4 Institute of Liver and Biliary Sciences, New Delhi, Delhi, India; FIOCRUZ, BRAZIL

## Abstract

**Background:**

Lack of awareness about viral hepatitis (VH) potentially predisposes the healthcare workers (HCWs) to a higher risk of infection and may in turn increase the risk of transmission of the infection to their families and in the community. Thus, combating VH, requires adequate and updated training to the HCWs. With this objective, Project PRAKASH designed a meticulously planned training program, aimed to assess the effect of a one-day training on VH among in-service nurses.

**Methods and material:**

The content and schedule of scientific sessions of the training program were decided by subject experts to improve knowledge, attitude and practice(KAP) related to VH among in-service nurses. A 54-item questionnaire divided into four domains: Transmission and Risk Factors; Prevention; Treatment; Pathophysiology and Disease Progression were used to assess the KAP related to VH. The questionnaire consisted of four sections: demographic details, knowledge(30-items), attitude(12-items) and practice(12-itmes) with a total score of 30, 60 and 24 respectively in each section. The pre-post knowledge assessment was done and impact assessment survey was undertaken among the participants who completed six months post-training period. Paired-t-test was used to assess the effect of training on knowledge using SPSSv-22.

**Results:**

A total of 5253 HCWs were trained through 32 one-day trainings, however data for 4474 HCWs was included in final pre-post knowledge analysis after removing the missing/incomplete data. Mean age of participants was 33.7±8.4 with median experience of 8(IQR: 3–13). Mean improvement in knowledge score was found to be significant (p<0.001) with mean knowledge score of 19.3±4.4 in pre-test and 25.7±3.9 in the post-test out of 30. Impact assessment survey suggested change in attitude and practice of HCWs.

**Conclusion:**

The one-day training programs helped the in-service nurses to enhance their knowledge related to viral hepatitis. The study provided a roadmap to combating viral hepatitis through health education among HCWs about viral hepatitis.

## Introduction

Healthcare workers (HCW) are at increased risk of occupational exposure to several blood-borne infections such as Human Immunodeficiency Virus (HIV), Hepatitis B virus (HBV), and Hepatitis C virus (HCV) by virtue of their professional exposure to blood and other bodily fluids during the close contact with patient [1, 2]. Globally, around three million (two million of HBV and 0.9 million of HCV) HCWs are exposed with HBV and HCV viruses, of which approximately 85000 (70,000 HBV infection and 15000 HCV infection) contract the infection annually [3] Further 90% these infections are reported from developing countries [2], mainly attributable to overcrowded hospitals [4], inadequate supply of basic personal protective equipment (PPE) [5], re-utilization of used needles [6], unsafe injection practices [2], improper disinfection practices, inadequate knowledge and neglecting attitude towards availing post-exposure prophylaxis [7] and limited or incorrect awareness about risk of exposure to blood and body fluids [8, 9].

Moreover, despite availability of safe and effective vaccine for Hepatitis B for more than three decades, coverage of vaccination remains low among HCWs for various reasons [10]. Lack of awareness, risk assessment associated with Hepatitis B infection, low priority given by health management and policy makers and negligence towards getting themselves vaccinated are few reasons for incomplete or partial vaccination against HBV [11–13]. Prevalence of HBV in general population of India is 2.4% [14] whereas the prevalence of HBV in HCWs was found to be less than 1% [15–17]. Despite the low prevalence of HBV among Indian HCWs [15–17] (2.4%) they can serve as super spreaders for these deadly infections in the community as well as to their family members [18–21]. An incidence of HBV outbreak has been reported from a town in Gujarat attributable to unsafe injection practices followed by HCWs where 664 cases were identified to be HBV positive [20]. In addition to this, lack of awareness has led to formation of stigma among HCWs while treating viral hepatitis patients which could jeopardize the management of patients. Thus, inadequate knowledge and awareness about viral hepatitis has drastically affected attitude and practice of the HCWs [22].

Contemplating this, Institute of Liver and Biliary Sciences, conceived a training program for in-service HCWs particularly nurses under Project PRAKASH (PRogrammed Approach to Knowledge And Sensitization on Hepatitis), a prerequisite steps towards combatting viral hepatitis as per 3.3 goal of Sustainable Development Goals (SDG) of 2030. The objective of the program was to impart the latest knowledge about prevention and management of viral hepatitis among nursing professionals across the country. The aim of the present study is to assess the effectiveness of the one-day training program on viral hepatitis through pre-post knowledge assessment survey. The study also aimed to explore the factors associated with knowledge gain in one-day training program on viral hepatitis and also to assess the impact of one-day training program on viral hepatitis on their attitude and practice at least after six months.

## Materials and methods

A one-day training program on Viral Hepatitis titled ‘Hepatitis Induction Program’ was conducted for a period of two years (January 2018 till January 2020) on a regular basis with an aim to impart up to date knowledge to nursing professionals in management of Viral Hepatitis. These trainings were organised at Institute of Liver and Biliary Sciences (ILBS), a tertiary care hospital with specialized in liver care under Government of National Capital Territory of Delhi. The training program was conducted in five phases which are described as below:

### Phase 1: Preparation of training program

The first phase of the program included planning and preparation of the training program which included finalisation of scientific agenda, speakers, and reference material. Certified Nurse Educator (CNE) accreditation was obtained from Delhi Nursing Council. This phase also comprised of preparation and validation of Knowledge, Attitude and Practice (KAP) and Pre-Post assessment questionnaire. Following which liasoning and engaging with various colleges was accomplished by circulating the brochure of the training program through various modes and mediums with the principals, nursing officers, trainers, educators, nursing supervisors and faculties associated with college of nursing for registration of the participants. The training program provided online as well as offline provision for registration.

### Phase 2: Pre knowledge, attitude and practice assessment

The trainings of nursing professionals were undertaken through physical mode at APJ Abdul Kalam Auditorium, ILBS, New Delhi. An online link to KAP questionnaire designed in SurveyMonkey was shared before the training through the registered mobile number of the participants. The 54-item KAP questionnaire consisted of four sections: demographic details, knowledge (30-items), attitude (12-items) and practice (12-items) (S1 Appendix).

Section A consisted of demographic details which included variables like sex, age and number of years practising as HCWs. Section B entailed 30 multiple choice questions assessing knowledge of the participants; one mark was awarded for every correct response thus, a total of 30 marks were allotted to this section. Knowledge section was further divided into four major domains: (i) Transmission and Risk Factors; (ii) Prevention; (iii) General & Treatment Related & (iv) Pathophysiology & Disease Progression. Section C assessed 12 questions related to attitude of HCWs towards viral hepatitis. The attitude questions were designed on five point-Likert scale for participants to choose from how much they agree or disagree with a particular statement. For positive questions ‘strongly agreed’ was coded as five; ‘agreed’ as four; ‘neutral’ as three, ‘disagree’ as two and ‘strongly disagree’ as one. For negative questions coding was done in an opposite way with ‘strongly disagreed’ as five to ‘strongly agreed’ as one. The total score of the attitude questions was 60. Section D consisted of 12 questions related to practice. Always, Sometimes and Never was the provided options for the practice section. In a positive statement Always was coded as two, sometimes as one and never as zero whereas in a negative statement Always was coded as zero, never as two. The total score of the practice section was 24.

### Phase 3: Training program

**Fig 1:**
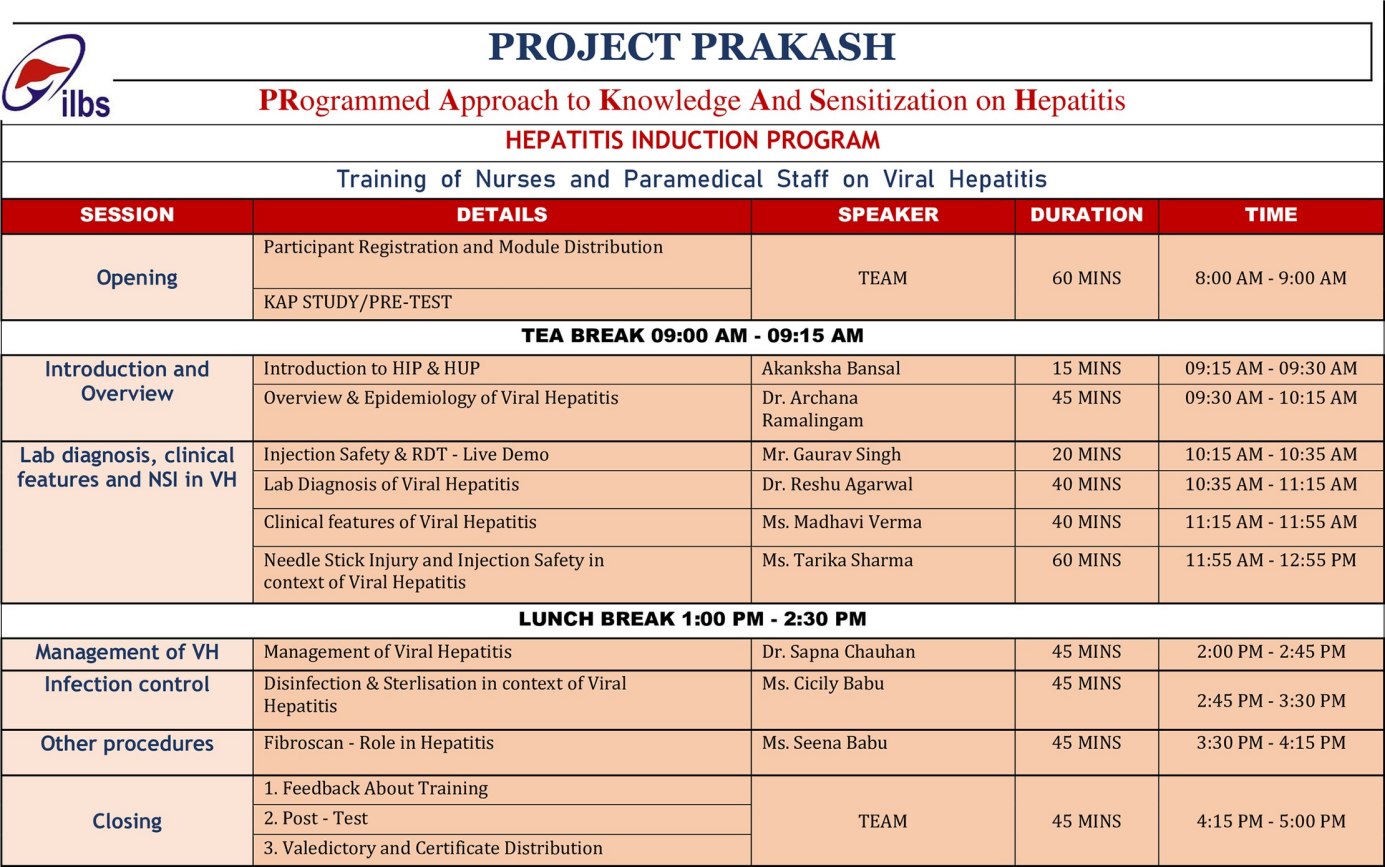
Agenda of the one-day training program for nurses on viral hepatitis under project PRAKASH.

The scientific training related to viral hepatitis were imparted by the subject-expert through face-to-face medium covering six important scientific topics with session duration ranging from 45 minutes to one hour each (Fig 1). Following the end of the session, session-experts addressed the queries of the participants. All participants who attended full day training program were given ‘Certificate of Participation’ accredited with 8-credit hours by Delhi Nursing Council.

### Phase 4: Post test: Scoring of questions

Following the end of the scientific sessions, link of Post-test questionnaire was sent to all participants via text message on their registered mobile number. The online link to post knowledge assessment was prepared using SurveyMonkey platform. It consisted of 30 questions related to knowledge about viral hepatitis as similar to pre-knowledge assessment (Knowledge section of KAP).

### Phase 5: Impact assessment

In addition to pre-post, a small impact assessment survey (IAS) was also undertaken in a sub-population of the participants. The IAS aimed at assessing the change in the attitude and practice with respect to routine/clinical practice on viral hepatitis. The Impact assessment Survey (IAS) questionnaire was sent to only those participants who have completed at least six months post-training period. A total of 10% response was expected to be collected through impact assessment survey.

This research was undertaken as a part of outreach activity. However ethical clearance was obtained from Institute Ethics Committee (IEC) of ILBS via letter F.37/(1)/9/ILBS/DOA/2020/20217/78 dated 1^st^ March 2021 for analyzing the data. Informed consent of the participants was obtained at the time of data collection as the first page of the survey included a brief paragraph about informed consent. Furthermore, the participants had the right to withdraw from the study at any stage. The identification details were made anonymous by providing unique identity numbers to the participants and the results were kept confidential and wasn’t shared with anyone apart the research team.

The data was extracted in MS excel cleaned. The continuous variable was presented as mean and standard deviation (SD) or median and Inter quartile range (IQR) as appropriate. The categorical variable was presented as frequency and percentages. Paired t-test was used to assess the difference in pre and post knowledge assessment. For the purpose of further analysis the age was categorised into two categories as less than 30 years and 30 years and above [23, 24]. Similarly, the years of experience was grouped into two as less than five years and five years and more [25]. For performing the requisite analysis, knowledge score was divided as poor-to-moderate (<75%) and Good (≥75%) [26, 27]. Chi square or student t-test was used to explore the association between knowledge, attitude and practice scores and demographic characteristics. The multivariate analysis was performed and Odds Ratio (OR) with 95% Confidence Interval (CI) was presented. For the purpose of logistic regression a new category was created with knowledge gain more than six and less than six unit, decided by the median change in knowledge [28]. The analysis was performed in IBM-SPSS version 22.

## Results

A total of 32 one-day training programs were organised for nursing professionals at ILBS from institutions across 12 states of India were trained about prevention and management of viral hepatitis. Though a total of 5253 HCWs attended the training, we had pre-knowledge assessment data for 4920 participants and 4474 responses for post-knowledge assessment with a response rate of 85.1% as pre and post knowledge assessment was completely voluntary. Considering this, a total of 4920 was considered for KAP assessment and 4474 participants were considered in the final pre-post analysis.

### Knowledge, attitude and practice assessment

A total of 4920 participants were considered in KAP assessment with mean age of 33.62±8.47 years and 64.8% belonging to age group 30 and above. Among the total participants, one-third participants were females (n = 3594, 73.0%) and approximately 95.4% of them were working in healthcare settings based in Delhi. The median years of experience as HCW was 8 years (IQR: 3–13) among the training participants with 66.8% had experience of 5 or more years.The majority of participants (88.4%) were working in government healthcare facility (Table 1).

**Table 1:** General characteristics of the participants in pre (n = 4920) and post assessment (N = 4474).

Characteristics	Pre- assessment	Post assessment.
	n (%)	n (%)
Mean Age (SD) years	33.62 (8.47)	33.30 (8.02)
**Gender**		
Male	1326 (27.0)	1221 (27.3)
Female	3594 (73.0)	3253 (72.7)
**Type of Facility**		
Government	4348 (88.4)	3961 (88.5)
Private	572 (11.6)	513 (11.5)
**States**		
Delhi	4696 (95.4)	4256 (95.1)
Outside Delhi	224 (4.6)	218 (4.9)
Median Years of		
Experience (IQR)	8 (3–13)	8 (3–13)

SD: Standard deviation; IQR: Interquartile range.

The mean knowledge, attitude and practice scores were found to be 19.23±4.53, 46.71±8.80, 18.65±3.67 out of total score of 30, 60 and 24 respectively. Correlation statistics between knowledge, attitude and practice score stated a positive correlation between knowledge and attitude (r = 0.38, p<0.001), knowledge and practice (r = 0.22, p<0.001), attitude and practice (r = 0.42, p<0.001). Approximately, only 22.78% of the HCWs were having good knowledge score while remaining had poor-to-moderate knowledge score.

Further, on multivariate analysis of demographic factors with KAP score demonstrated age, gender and type of facility was found to be significantly associated (p<0.05) with baseline knowledge, attitude and practice. In addition to this, years of experience was found to significantly associated with knowledge score only and location of healthcare facility was found to be significantly associated with attitude score (p<0.05) as described in Table 2.

**Table 2:** Association of demographic characteristics with knowledge, attitude and practice score (n = 4920).

Demographic Characteristics	Mean Knowledge Score (SD)	p-value	Mean Attitude Score (SD)	p-value	Mean Practice Score (SD)	p-value
**Age Category**						
Less than 30 years	19.80 (4.44)	<0.001	47.29 (7.72)	<0.001	18.79 (3.43)	0.046
30 years and above	18.92 (4.55)		46.40 (9.32)		18.57 (3.80)	
**Gender**						
Male	19.48 (4.75)	0.018	46.15 (8.82)	0.007	19.00 (3.55)	<0.001
Female	19.13 (4.44)		46.92 (8.78)		18.52 (3.72)	
**Experience Category**						
Less than 5 years	19.62 (4.52)	<0.001	46.80 (7.68)	0.636	18.76 (3.46)	0.157
5 years and above	19.03 (4.52)		46.67 (9.30)		18.60 (3.78)	
**Facility Type**						
Private	17.67 (4.56)	<0.001	45.41 (6.97)	<0.001	18.94 (3.56)	0.048
Government	19.43 (4.49)		46.89 (9.00)		18.61 (3.69)	
**Location**						
Outside Delhi	18.68 (4.65)	0.063	45.48 (8.06)	0.032	18.80 (3.60)	0.526
Delhi	19.25 (4.52)		46.77 (8.83)		18.64 (3.68)	

SD: Standard deviation.

### Pre and post knowledge assessment

A total of 4474 participants were considered in the analysis of pre-post knowledge assessment. The mean pre-knowledge assessment of participants was found to be 19.3±4.4 whereas the post-knowledge assessment was 25.7±3.9 out of total score of 30; this difference of 6.34 units (95% CI: 6.22–6.47) in the knowledge scores was found to be significant with p-value<0.001. The percentage improvement in knowledge ranged from 2.1% - 347.3% (Refer to S1 Table). The domain wise mean pre-knowledge score was 6.94±1.72 out of 9 in transmission and risk factor associated domain, 4.29±1.35 out of 6 in prevention domain, 5.82±1.55 out of 9 in general and treatment related and 2.30±1.31 out of 6 in pathophysiology and disease progression domain. Similarly, post-assessment knowledge domains are described in Table 3. Significant mean difference was observed in pre and post knowledge assessment with respect to domains mentioned above.

**Table 3:** Domain-wise pre and post knowledge assessment score (n = 4474).

Domains	Mean Pre Assessment knowledge	Mean Post Assessment knowledge	Mean difference (95% CI)	T-test	p-value
	Score (SD)	Score (SD)			
Transmission & risk factor (K2, K5, K7, K10, K19, K20, K25, K27, K29)	6.94 (1.72)	8.07 (1.11)	1.13 (1.08–1.18)	44.95	<0.001
Prevention (K14, K15, K16, K24, K26, K30)	4.28 (1.35)	527 (1.06)	0.98 (0.94–1.02)	49.34	<0.001
General and Treatment related (K1, K3, K11, K12, K13, K21, K22, K23, K28)	5.81 (1.54)	7.54 (1.42)	1.72 (1.66–1.77)	64.40	<0.001
Pathophysiology and disease progression (K4, K6, K8, K9, K17, K18)	2.30 (1.30)	4.81 (1.26)	2.52 (2.46–2.56)	104.03	<0.001
Total Score	19.34 (4.39)	25.69 (3.89)	6.34 (6.22–6.47)	97.61	<0.001

SD: Standard deviation; CI: Confidence interval.

The unadjusted and adjusted multivariate analysis with median change in knowledge and demographic variables suggested gender and type of facility were found to significantly associated with median gain in knowledge. The female participants had 1.25 odds (95% CI: 1.09–1.43, P <0.001) of median gain in knowledge as compared to their male counterparts after adjusting for other variables. Similarly, participants working in government setting had 1.38 odds (95% CI: 1.14–1.67, P = 0.001) of median gain in knowledge when compared to participants working in private healthcare facility after adjusting for various demographic factors. The age and years of experience was not found to be significantly associated with median gain in knowledge as described in Table 4.

**Table 4:** Association of demographic characteristic with median change in knowledge^a^ and demographic factors (N = 4474).

Demographic characteristic	OR (95% CI)	p-value	aOR (95% CI)	p-value
**Age**				
>30 years	**Ref**		**Ref**	
<30 years	0.98 (0.87–1.10)	0.746	0.97 (0.83–1.14)	0.734
**Gender**				
Male	**Ref**		**Ref**	
Female	1.28 (1.12–1.46)	<0.001	1.25 (1.09–1.43)	0.001
**Type of facility**				
Private	**Ref**		**Ref**	
Government	1.39 (1.16–1.68)	<0.001	1.38 (1.14–1.67)	0.001
**Experience**				
< 5 years	**Ref**		**Ref**	
> 5 years	1.04 (0.92–1.18)	0.502	1.08 (0.91–1.28)	0.370

^a^ Median change in knowledge is considered at 6 units increase in knowledge post training.

P value of the model <0.001; Psuedo R^2^: 0.004. OR: odds ratio; aOR: adjusted odds ratio; CI: confidence interval; Ref: Reference.

### Impact assessment

A total of 5253 participants attended the Hepatitis Induction program for nursing professionals, out of which 779 participants were excluded since they haven’t responded to either pre or post test resulting in 4474 participants. Further, 2615 participants were eligible as they have completed six months of post training period when the impact assessment survey was circulated. A link to impact assessment survey was shared with participants who have completed at least six months post-training period which yielded a response of 23.8% with 623 responses. Out of which 534 (20.4%) responses were included in the analysis after removing the duplicates and incomplete entries. The impact assessment survey indicated that 82% of the participants (n = 438) initiated the use of Personal Protective Equipment’s (PPEs) on a regular basis post attending training. It was found that 97.9% (n = 523) respondents were following the Injection safety protocols on a regular basis, post training. In addition, 79.8% (n = 426) participants have started advising HBV and HCV patients and their family members about screening of viral hepatitis on regular basis. Approximately, 83% (n = 443) participants started advising high risk patients and their relatives about the need of HBV vaccination on regular basis (Table 5).

**Table 5:** Responses of impact assessment survey (N = 534).

Impact Assessment Survey Questions	Never n(%)	Sometimes n(%)	Always n(%)
Started following universal precautions	3 (0.56)	41 (7.68)	490 (91.76)
Started using Personal Protective Equipment’s (PPE)	3 (0.56)	93 (17.42)	438 (82.02)
Started following injection safety protocols	1 (0.19)	10 (1.87)	523 (97.94)
Started reporting Needle Stick Injuries (NSI)	7 (1.31)	43 (8.05)	484 (90.64)
Started advising high risk patients and relatives about Hepatitis B and C testing	6 (1.12)	102 (19.10)	426 (79.78)
Started advising high risk patients and relatives about Hepatitis B vaccination	13 (2.43)	78 (14.61)	443 (82.96)
Started advising pregnant females for HbsAg testing	11 (2.06)	43 (8.05)	480 (89.89)

## Discussion

The inadequate knowledge among HCWs has resulted in inappropriate practices which in turn led to viral hepatitis outbreaks in communities [20, 21]. Further, inappropriate knowledge, resulted in formation of stigma which could have jeopardize treatment of patients [22]. Therefore, there is a dire need to train and educate the HCWs against viral hepatitis to combat it by 2030 as proposed by SDG 3.3 goal. Envisioning this, a training program was apprehended for in-service HCWs particularly nurses under Project PRAKASH. The aim of the present study was to assess the effectiveness of the one-day training program on knowledge on viral hepatitis through pre-post assessment and also to explore the impact of training on their attitude and practice at least after six months of training.

The current study suggested that HCWs had a mean knowledge score of 19.23±4.53 with 77.2% of the participants falling in poor-to-moderate category with respect to knowledge related to viral hepatitis. The knowledge score of the present study were in line with previous studies from developing countries [29, 30], this is mainly attributable to the ignorance and less importance given to viral hepatitis among physicians especially in developing countries [31, 32]. Further, younger participants (19.80±4.44) were found to be more aware about viral hepatitis as compared to their older counterparts (18.92±4.55, p<0.001) as observed in several other studies assessing the knowledge [33]. This could be due to fact that younger age group have recently completed their academic education and could recall the concepts better as compared to older HCWs [34, 35]. Moreover, same explanation can be considered for better knowledge among HCWs having less than five years of experience (19.62±4.52) when compared with more experienced HCWs (19.08±4.52,p<0.001).

This KAP study re-established a positive correlation between knowledge, attitude and practice score, similar to previous studies indicating association of better knowledge with positive attitude and better medical practices [36, 37]. The study highlighted despite low knowledge levels, positive attitude and good medical practice was reported among the participants which could be a result of social desirability response. This findings of the present study were supported by a study from Ethiopia, which indicating good knowledge but poor practice related to HBV, which resulted in intermediate prevalence of HBV in Ethiopian HCWs [38]. Another study from Cameroon reported that despite the presence of good knowledge, high prevalence of HBsAg positivity was observed among HCWs [39]. Thus, it is equally important to have good knowledge as well as follow good medical practices for reducing the burden of viral hepatitis among HCWs. However, the present study didn’t study the prevalence of HBV and HCV, and hence couldn’t relate knowledge, attitude and practice related to viral hepatitis to its prevalence.

With respect to domain wise knowledge score, the domain concerned with transmission and prevention of the viral hepatitis was found to have maximum score as compared to domains associated with treatment and pathophysiology and disease progression associated with viral hepatitis. Previous study has reported higher knowledge score in prevention domain as result of repeated educational course during work years. However, our study showed lower knowledge score in treatment domain which could be mainly explained by difficulty level of questions in both the studies [40].

Project PRAKASH educated and empowered a total of 5253 nursing professionals from 292 institutions across 12 states through 32-one-day trainings conducted over two years. The one-day training with a combination of theoretical as well as practical sessions helped the in-service nurses to enhance their knowledge related to viral hepatitis as observed from mean post-knowledge score of 25.69±3.89 which was found to be statistically more when compared with pre-knowledge score of 19.34±4.39. Our study was in line with previous studies which demonstrated improvement in knowledge score followed by extensive training on Indian HCWs [41–43]. The percentage improvement in knowledge was found to be more in questions where understanding and practical application of concepts was required. Further, not much improvement was observed in fact based questions because they were easy and most of the participants responded correctly in pre-knowledge assessment, thus there was minimal room for improvement.

The one-day training resulted in modification in attitude and practices of HCWs as observed from the results obtained from impact assessment survey. Considerable positive change was observed in increased use of PPEs, following of injection safety protocols and increased vigilance towards needle stick injury. Furthermore, the participants became more concerned about their hepatitis B vaccination status as they got themselves vaccinated and also checked their antibody titre levels. In addition to this, they started counselling the family members of Hepatitis B and Hepatitis C positive individuals to get themselves screened and vaccinated. Since, there was no such study among HCWs assessing the impact on attitude and practices, six months after the training, authors could not correlate their findings to other studies. However, better knowledge has resulted in increased vaccination coverage among HCWs as reported by other studies [13, 44, 45], thus these modification in attitude and practices can be attributable to knowledge gained through training.

In this novel study, for the first time it has been demonstrated that HCWs can be empowered against viral hepatitis by providing adequate training which is even reflected in their attitudes and practices even after six months of the training. The limitation of the present study is that it was not able to collect post-test responses from approximately 15% of the participants and also it can’t be assessed whether non-participation was unintentional or intentional because of expecting low score. However, there was no significant difference observed in demographic characteristics of the participants of who filled and who didn’t filled post knowledge assessment. Also, the tool employed to assess KAP was not a validated tool. Moreover, all the information that was collected by the participant was not necessarily reflection of the knowledge and attitude level of entire nursing fraternity. The study could have also suffered a response-shift bias because of its pre-post design [46]. There could have been selection bias in impact assessment survey as the participants who filled the survey were based on voluntary participation. Thus, alteration of attitude and practice might not be representative of all nursing professionals in the country.

Despite these inherent limitations, there have been many unique aspects and strengths of the study. Firstly, to the best of our knowledge, this is one of the biggest and pioneer study which had trained more than 5000 HCWs about prevention and management of viral hepatitis. Moreover, the study was able to provide and follow a sub-sample of nurses to assess the impact of one-day training on the clinical practices and personal protection. The one-day training program was able to increase knowledge of HCWs and motivated them to modify their attitude and practices. This will not only help in better management of viral hepatitis patients but will also be beneficial in protecting themselves with occupational exposure to viral hepatitis. In light of the study findings, comprehensively designed one-day training program provided a roadmap for combating viral hepatitis through health education among HCWs about viral hepatitis. Thus, training and awareness program can help in addressing a common health problem by targeting high-risk group of HCWs, which can have a significant influence on reducing the burden of viral hepatitis.

## Conclusions

Comprehensively designed one-day training program was able to improve the knowledge of the healthcare workers and also reflected change in attitude and practices towards viral hepatitis. Therefore, there should be a provision of basic or refresher training related to viral hepatitis for healthcare workers, which will not only update them about the disease but will also motivate them to have positive attitude towards patients and to follow good medical practice with standard precautions. Collectively these efforts can help in combating viral hepatitis by 2030.

## Supporting information

S1 Dataset (XLS)

S1 File KAP questionnaire (nurses). (PDF)

S1 Appendix Knowledge, attitude and practice questionnaire. (PDF)

S1 Table Responses of pre-post knowledge assessment question wise (N = 4474). (DOCX)

S2 Table Attitude related questions with percentage of responses. (DOCX)

S3 Table Practice related questions with percentage of responses. (DOCX)
