# Supplementary material for: Capacity building of healthcare workers: Key step towards elimination of viral hepatitis in developing countries
Source: PLoS One. 2021 Jun 24;16(6):e0253539. doi: 10.1371/journal.pone.0253539 (PMC8224969; doi:10.1371/journal.pone.0253539)
Supplement: S1 File — (PDF) [file pone.0253539.s002.pdf]

S1 File: KAP Questionnaire (Nurses)

A. Demographic Details of the Participants

Name:
Gender: Male Female
Age:
Name of the Hospital:
Location of working:
Type of Institute: Government Private
Number of Practicing Years:
Mobile Number:
Email Id:

B. Knowledge related questions

1. What is Hepatitis?
    A. Inflammation of Liver
    B. Inflammation of Lung
    C. Inflammation of Abdomen
    D. Inflammation of Brain

2. Hepatitis A is transmitted through?
    A. Contaminated food and water
    B. Eating raw shellfish
    C. Food Handler
    D. All of the above

3. What are the types of viral hepatitis known?
    A. Hepatitis A, B, C, D & E
    B. Hepatitis A, B, C & D
    C. Hepatitis A, B & C
    D. Hepatitis A & B

4. Acute Liver Failure especially in pregnant women is caused in which Hepatitis virus?
    A. Hepatitis A
    B. Hepatitis B
    C. Hepatitis C
    D. Hepatitis E

5. Following pose increased risk for hepatitis A and E:
    A. Poor sanitation
    B. Poor hygiene
    C. No access to safe water
    D. All of the above

6. Following are true about hepatitis A and E EXCEPT:
   A. Mostly spread by feco-oral route
   B. Hepatitis A vaccine is available in some countries
   C. Both hepatitis A & E can have chronic states
   D. Sanitation & hygiene can prevent occurrence of these infections

7. Hepatitis B is NOT transmitted by:
   A. Blood and blood products
   B. Hugging and kissing
   C. Sexual route
   D. Needle Stick Injury

8. Chronic viral hepatitis is hepatitis that lasts more than
   A. 15 days
   B. 1 month
   C. 3 months
   D. 6 months

9. Infection at what age can lead to maximum chance of chronicity for hepatitis B?
   A. Infancy
   B. 1-5 years
   C. >5 years
   D. All are same

10. Who is NOT at risk for Hepatitis B:
   A. Injection drug user
   B. Hemodialysis patient
   C. Person having multiple sexual partners
   D. Person consuming street food

11. To clean blood spills from an HBV infected person what should be used?
   A. 1:4 hypochlorite solution
   B. 1:10 hypochlorite solution
   C. Plain water
   D. Normal saline solution

12. A HBV infected person can
   A. Donate blood
   B. Donate organ
   C. Donate both
   D. Donate none

13. Following are true for HBV infection treatment:

A. Patient should be monitored regularly
B. Acute HBV infection may resolve spontaneously
C. There is no cure for chronic HBV infection
D. All of the above

14. Following are true about HBV vaccine EXCEPT:
A. Vaccine is available in India
B. All health care workers should be vaccinated
C. HBV vaccine should not be given to a newborn of an HBV positive mother
D. Primary vaccination of infants consists of 3 doses

15. Following strategies can be used for preventing HBV infection EXCEPT:
A. Immunization
B. Sanitation and hygiene
C. Screening of pregnant women for HBV infection
D. Safe sex

16. A child born to a HBV infected mother should receive
A. HBIG at birth
B. HBV vaccine within 12-24 hours of birth
C. Both
D. None

17. All of the following are TRUE about Hepatitis C EXCEPT:
A. Caused by Hepatitis C virus
B. Have highest chronicity potential
C. Is curable
D. None of the above

18. The following can be caused as a sequelae of HCV infection
A. Primary Liver Cancer
B. Cirrhosis of Liver
C. Both
D. None

19. HCV is transmitted by all EXCEPT:
A. Contaminated food and water
B. Sexual contact
C. Sharing needles
D. Needle stick injury

20. Following people are at risk for HCV infection:
A. Injection drug user
B. Hemodialysis patient

C. HIV patient
D. All of the above

21. Following are true about HCV infection EXCEPT:
A. 70-80% acute HCV infections have no symptoms
B. A person can spread the virus even if he/she has no symptoms
C. Can be acquired by a person getting tattoo in a place with poor infection control practices
D. Risk of transmission by sexual route is extremely high

22. Following is NOT true about HCV treatment:
A. Drugs are available for treatment of HCV
B. Blood tests for virus needs to be done before and after completion of treatment
C. Treatment duration is for 12-24 weeks
D. Lifelong treatment is necessary for HCV

23. Following statement is true about HCV infection
A. One cannot donate organ or blood if he/she is HCV infected
B. There is an effective vaccine against HCV
C. Sanitation and food hygiene is necessary for prevention
D. Needle Stick Injury cannot cause HCV infection

24. Following are the personal protective equipment's EXCEPT:
A. Gloves
B. Ear Plugs
C. Mask
D. Gown

25. Needle stick injury can cause the following infection
A. HCV
B. HBV
C. HIV
D. All

26. The following needs to be done after a needle stick injury EXCEPT
A. Squeeze out as much blood as possible from the site
B. Report the Needle Stick Injury
C. Take post-exposure prophylaxis
D. Wash the area with soap and water

27. The following type of hepatitis is food and water borne:
A. Hepatitis A
B. Hepatitis B
C. Hepatitis C
D. Hepatitis D

28.The following statements are true EXCEPT:

A. Hepatitis A has an effective vaccine but is not routinely given in India
B. Hepatitis E may cause fulminant hepatitis in pregnant women
C. Hepatitis B vaccine is incorporated in NIS
D. Hepatitis C vaccine is effective in preventing infection

29. All of the following can be transmitted through infected blood EXCEPT:

A. Hepatitis B
B. Hepatitis C
C. Both
D. None

30. The route of administration of hepatitis B vaccine is:

A. Intramuscular
B. Intradermal
C. Subcutaneous
D. Intravenous

C. Attitude related questions

---

1. I think I am not at risk for getting Hepatitis
A. Strongly Agree B. Agree C. Neutral D. Disagree
E. Strongly Disagree

2. Hepatitis B vaccine is safe and effective
A. Strongly Agree B. Agree C. Neutral D. Disagree
E. Strongly Disagree

3. Needle-stick Injury at workplace should be immediately reported to the concerned authority
A. Strongly Agree B. Agree C. Neutral D. Disagree
E. Strongly Disagree

4. Patient with Hepatitis should have the same rights as others
A. Strongly Agree B. Agree C. Neutral D. Disagree
E. Strongly Disagree

5. Hepatitis B and C patients should be isolated from the society
A. Strongly Agree B. Agree C. Neutral D. Disagree
E. Strongly Disagree

6. I have no concern of being infected with HBV while caring for a patient with HBV infection
A. Strongly Agree B. Agree C. Neutral D. Disagree

E. Strongly Disagree

7. While caring for a patient with HBV infection, it is my responsibility to follow universal precautions
A. Strongly Agree B. Agree C. Neutral D. Disagree
E. Strongly Disagree

8. Changing of gloves during blood collection and tests is waste of time
A. Strongly Agree B. Agree C. Neutral D. Disagree
E. Strongly Disagree

9. All patients should be tested for HBV before they receive health care
A. Strongly Agree B. Agree C. Neutral D. Disagree
E. Strongly Disagree

10. Following the infection control guidelines will protect the healthcare worker from being infected with HBV at work
A. Strongly Agree B. Agree C. Neutral D. Disagree
E. Strongly Disagree

11. Needle should be recapped/bent after use
A. Strongly Agree B. Agree C. Neutral D. Disagree
E. Strongly Disagree

12. I would refer a patient to appropriate health facility immediately if s/he has symptoms of Hepatitis B
A. Strongly Agree B. Agree C. Neutral D. Disagree
E. Strongly Disagree

D. Practice related questions

---

1. Do you use gloves for PHLEBOTOMY procedures?
A. Yes B. No C. Not Sure

2. Do you make sure that you use a new/sterile syringe for any procedure?
A. Yes B. No C. Not Sure

3. Have you been screened for Hepatitis B?
A. Yes B. No C. Not Sure

4. Have you got yourself vaccinated against Hepatitis B?
A. Yes B. No C. Not Sure

5. Do you avoid recapping/bending the needle after use?
A. Yes B. No C. Not Sure

6. Do you dispose sharps in puncture proof container after use?

A. Yes B. No C. Not Sure

7. Would you educate your patient to ask barber to change blade/or for safe equipment's for ear and nose piercing?
   A. Yes B. No C. Not Sure

8. Would you educate your patient to ask for screening of blood before receiving blood transfusion?
   A. Yes B. No C. Not Sure

9. Would you advise a patient who has just got diagnosed with Hepatitis B to go for further investigation and treatment?
   A. Yes B. No C. Not Sure

10. Would you advise a patient diagnosed with Hepatitis B to share food/utensils/water etc. with others?
    A. Yes B. No C. Not Sure

11. Would you advise a patient diagnosed with Hepatitis B to avoid meeting with people?
    A. Yes B. No C. Not Sure

12. Have you ever participated in health education program related to Hepatitis B?
    A. Yes B. No C. Not Sure
