# Supplementary material for: Capacity building of healthcare workers: Key step towards elimination of viral hepatitis in developing countries
Source: PLoS One. 2021 Jun 24;16(6):e0253539. doi: 10.1371/journal.pone.0253539 (PMC8224969; doi:10.1371/journal.pone.0253539)
Supplement: S1 Table — (DOCX) [file pone.0253539.s004.docx]

**S1 Table: Responses of Pre-post Knowledge assessment Question wise (N=4474)**

|  | **Knowledge related Questions** | **Pre-score** | **Post score** | **Knowledge improvement (%)** |
| --- | --- | --- | --- | --- |
| K1 | What is Hepatitis? | 4358 (97.4) | 4449 (99.4) | 2.09 |
| K2 | Hepatitis A is transmitted through? | 2185 (48.8) | 3237 (72.4) | 48.15 |
| K3 | What are the types of viral hepatitis known? | 3446 (77) | 4366 (97.6) | 26.70 |
| K4 | Acute Liver Failure especially in pregnant women is caused in which Hepatitis virus? | 1237 (27.6) | 3712 (83) | 200.08 |
| K5 | Following pose increased risk for hepatitis A and E | 3872 (86.5) | 4302 (96.2) | 11.11 |
| K6 | Following are true about hepatitis A and E EXCEPT | 1990 (44.5) | 3572 (79.8) | 79.50 |
| K7 | Hepatitis B is NOT transmitted by | 4130 (92.3) | 4394 (98.2) | 6.39 |
| K8 | Chronic viral hepatitis is hepatitis that lasts more than | 2397 (53.6) | 4271 (95.5) | 78.18 |
| K9 | Infection at what age can lead to maximum chance of chronicity for hepatitis B? | 771 (17.2) | 3449 (77.1) | 347.34 |
| K10 | Who is NOT at risk for Hepatitis B | 3692 (82.5) | 4308 (96.3) | 16.68 |
| K11 | To clean blood spills from an HBV infected person what should be used? | 2948 (65.9) | 4052 (90.6) | 37.45 |
| K12 | A HBV infected person can | 4174 (93.3) | 4325 (96.7) | 3.62 |
| K13 | Following are TRUE for HBV infection treatment | 2309 (51.6) | 3296 (73.7) | 42.75 |
| K14 | Following are true about HBV vaccine EXCEPT | 3339 (74.6) | 3838 (85.8) | 14.94 |
| K15 | Following strategies can be used for preventing HBV infection EXCEPT | 2871 (64.2) | 3906 (87.3) | 36.05 |
| K16 | A child born to a HBV infected mother should receive | 2304 (51.5) | 3245 (72.5) | 40.84 |
| K17 | All of the following are TRUE about Hepatitis C EXCEPT | 1159 (25.9) | 2642 (59.1) | 127.96 |
| K18 | The following can be caused as a sequelae of HCV infection | 2729 (61) | 3891 (87) | 42.58 |
| K19 | HCV is transmitted by all EXCEPT | 3585 (80.1) | 4227 (94.5) | 17.91 |
| K20 | Following people are at risk for HCV infection | 3987 (89.1) | 4322 (96.6) | 8.40 |
| K21 | Following are true about HCV infection EXCEPT | 1772 (39.6) | 3043 (68) | 71.73 |
| K22 | Following is NOT true about HCV treatment | 2087 (46.6) | 3663 (81.9) | 75.52 |
| K23 | Following statement is TRUE about HCV infection | 2819 (63) | 3288 (73.5) | 16.64 |
| K24 | Following are the personal protective equipment’s EXCEPT | 4120 (92.1) | 4341 (97) | 5.36 |
| K25 | Needle stick injury can cause the following infection | 4056 (90.7) | 4348 (97.2) | 7.20 |
| K26 | The following needs to be done after a needle stick injury EXCEPT | 3409 (76.2) | 4008 (89.6) | 17.57 |
| K27 | The following type of hepatitis is food and water borne | 3802 (85) | 4355 (97.3) | 14.54 |
| K28 | The following statements are true EXCEPT | 1657 (37) | 3276 (73.2) | 97.71 |
| K29 | All of the following can be transmitted through infected blood EXCEPT | 1730 (38.7) | 2593 (58) | 49.88 |
| K30 | The route of administration of hepatitis B vaccine is | 3600 (80.5) | 4201 (93.9) | 16.69 |
