# Supplementary material for: Capacity building of healthcare workers: Key step towards elimination of viral hepatitis in developing countries
Source: PLoS One. 2021 Jun 24;16(6):e0253539. doi: 10.1371/journal.pone.0253539 (PMC8224969; doi:10.1371/journal.pone.0253539)
Supplement: S2 Table — (DOCX) [file pone.0253539.s005.docx]

**S2 Table: Attitude related questions with percentage of responses**

|  | **Questions** | **Strongly Agree (n) (%)** | **Agree (n) (%)** | **Neutral (n) (%)** | **Disagree (n) (%)** | **Strongly Disagree (n) (%)** |
| --- | --- | --- | --- | --- | --- | --- |
| A1 | I think I am not at risk for getting Hepatitis | 762 (16) | 1390 (29) | 628 (13) | 1131 (24) | 869 (18) |
| A2 | Hepatitis B vaccine is safe and effective | 1655 (35) | 2675 (56) | 227 (5) | 202 (4) | 36 (1) |
| A3 | Needle-stick Injury at workplace should be immediately reported to the concerned authority | 3976 (83) | 741 (16) | 29 (1) | 29 (1) | 27 (1) |
| A4 | Patient with Hepatitis should have the same rights as others | 2922 (61) | 1542 (32) | 111 (2) | 146 (3) | 39 (1) |
| A5 | Hepatitis B and C patients should be isolated from the society | 197 (4) | 354 (7) | 253 (5) | 2140 (45) | 1861 (39) |
| A6 | I have no concern of being infected with Hepatitis B virus while caring for a patient with Hepatitis B virus infection | 348 (7) | 764 (16) | 499 (10) | 1830 (38) | 1334 (28) |
| A7 | While caring for a patient with Hepatitis B virus infection, it is my responsibility to follow universal precautions | 3919 (82) | 701 (15) | 32 (1) | 61 (1) | 43 (1) |
| A8 | Changing of gloves during blood collection and tests is waste of time | 423 (9) | 187 (4) | 39 (1) | 1246 (26) | 2900 (61) |
| A9 | All patients should be tested for Hepatitis B virus before they receive health care | 81 (2) | 385 (8) | 440 (9) | 1876 (39) | 2012 (42) |
| A10 | Following the infection control guidelines will protect the healthcare worker from being infected with Hepatitis B virus at work | 2798 (59) | 1749 (37) | 116 (2) | 96 (2) | 33 (1) |
| A11 | Needle should be recapped/bent after use | 577 (12) | 390 (8) | 72 (2) | 1192 (25) | 2567 (54) |
| A12 | I would refer a patient to appropriate health facility immediately if s/he has symptoms of Hepatitis B | 2652 (55) | 1828 (38) | 90 (2) | 146 (3) | 48 (1) |
