# Supplementary material for: Capacity building of healthcare workers: Key step towards elimination of viral hepatitis in developing countries
Source: PLoS One. 2021 Jun 24;16(6):e0253539. doi: 10.1371/journal.pone.0253539 (PMC8224969; doi:10.1371/journal.pone.0253539)
Supplement: S3 Table — (DOCX) [file pone.0253539.s006.docx]

**S3 Table: Practice related questions with percentage of responses**

|  | **Questions** | **Yes (n) (%)** | **Not Sure (n) (%)** | **No (n) (%)** |
| --- | --- | --- | --- | --- |
| P1 | Do you use gloves for PHLEBOTOMY procedures? | 4547 (95) | 157 (3) | 76 (2) |
| P2 | Do you make sure that you use a new/sterile syringe for any procedure? | 4759 (98) | 12 (1) | 29 (1) |
| P3 | Have you been screened for Hepatitis B? | 3808 (79) | 185 (4) | 806 (17) |
| P4 | Have you got yourself vaccinated against Hepatitis B? | 4360 (91) | 105 (2) | 335 (7) |
| P5 | Do you avoid recapping/bending the needle after use? | 3944 (82) | 57 (1) | 796 (17) |
| P6 | Do you dispose sharps in puncture proof container after use? | 4744 (98) | 15 (1) | 45 (1) |
| P7 | Would you educate your patient to ask barber to change blade/or for safe equipment’s for ear and nose piercing? | 4584 (96) | 94 (2) | 119 (2) |
| P8 | Would you educate your patient to ask for screening of blood before receiving blood transfusion? | 4660 (97) | 43 (1) | 93 (2) |
| P9 | Would you advise a patient who has just got diagnosed with Hepatitis B to go for further investigation and treatment? | 4708 (98) | 35 (1) | 59 (1) |
| P10 | Would you advise a patient diagnosed with Hepatitis B to share food/utensils/water etc. with others? | 2651 (55) | 346 (7) | 1796 (38) |
| P11 | Would you advise a patient diagnosed with Hepatitis B to avoid meeting with people? | 522 (11) | 51 (1) | 4225 (88) |
| P12 | Have you ever participated in health education program related to Hepatitis B? | 1992 (41) | 106 (2) | 2706 (57) |
